# Poor immune response to inactivated COVID-19 vaccine in patients with hypertension

**DOI:** 10.3389/fmed.2024.1329607

**Published:** 2024-05-02

**Authors:** Lei Yang, TingTing Zeng, Yang Li, Qiao Guo, DePeng Jiang

**Affiliations:** ^1^Department of Respiratory Medicine, The Second Affiliated Hospital, Chongqing Medical University, Chongqing, China; ^2^Department of Endocrinology, The Second Affiliated Hospital, Chongqing Medical University, Chongqing, China; ^3^Department of General and Practice, The Second Affiliated Hospital, Chongqing Medical University, Chongqing, China

**Keywords:** COVID-19, safety, immunogenicity, inactivated vaccine, hypertension

## Abstract

**Purpose:**

The safety and efficacy of vaccination in people with hypertension (HTN) is important. There are currently a few data on the immunogenicity and safety of inactivated SARS-CoV-2 vaccinations in hypertension patients.

**Methods:**

After receiving a two-dose immunization, 94 hypertension adult patients and 74 healthy controls (HCs) in this study, the evaluation included looking at antibodies (Abs) against receptor binding domain (RBD) IgG, SARS-CoV-2 neutralizing antibodies (NAbs), RBD-specific B cells, and memory B cells (MBCs).

**Results:**

There was no discernible difference in the overall adverse events (AEs) over the course of 7 or 30 days between HTN patients and HCs. HTN patients had lower frequencies of RBD-specific memory B cells and the seropositivity rates and titers of Abs compared with HCs (all, *p* < 0.05). HTN patients with cardiovascular and cerebrovascular conditions (CCVD) have lower titers of CoV-2 NAb than in HCs. The titers of both Abs in HTN declined gradually over time.

**Conclusion:**

Inactivated COVID-19 vaccinations were safe in hypertension patients; however humoral immune was limited, especially merged CCVD and declined gradually over time.

## Introduction

Since its discovery in 2019 (1), the severe acute respiratory syndrome coronavirus 2 (SARS-CoV-2) has continued to devastate public health across the world. Even though several antiviral drug and specific monoclonal antibodies are currently available for the therapy of COVID-19, immunization is still essential for preventing COVID-19. HTN has been shown to have a higher risk of acquiring severe COVID-19 (2–4), therefore, the SARS-CoV-2 vaccines may be advantageous for this particular population.

Currently, hypertension still poses a threat to global public health. Numerous epidemiological studies (5, 6) have shown that the prevalence of hypertension in China ranges from 23.2% to 44.7%. Most individuals with hypertension, in particular, have co-occurring cardiovascular and cerebrovascular conditions (CCVD).

Recent studies have shown that HTN patients are protected after receiving a full vaccination, but the immune response may be lower than in HCs (3, 7–9). However, according to a study, there is no correlation between Ab response and hypertension (10). Because of this, it is currently unclear whether HTN patients in China may get the inactivated COVID-19 vaccination without harm and elicit a powerful immune response.

Following vaccination, antibody-secreting cells (ASCs), which are found in bone marrow and MBCs, mediate chronic humoral immunity. When an infection recurs, MBCs multiply quickly differentiate into ASCs, which aids in the defense of the body (11, 12). However, there has been little information available about how RBD^+^-specific MBCs response in HTN.

Previous studies have shown that COVID-19 vaccines are safe and effective (13–15). Due to the lack of information from clinical research, doctors might not be able to address concerns of patients about the safety and efficacy of COVID-19 vaccines in the HTN populations. Therefore, we evaluate the safety and immunogenicity of inactivated vaccinations, and this trial enrolled 94 adult HTN patients and 74 adult HCs.

## Materials and methods

### Study design

Participants in this study were systematically recruited at the Second Affiliated Hospital of Chongqing Medical University between 6 May 2021 and 3 December 2021. The diagnosis of hypertension was made based on the guidelines as follows: adults with an average SBP ≥140 mmHg or DBP ≥90 mmHg, and all patients who had the condition stayed stable and had good blood pressure management due to oral antihypertensive medications.

The key inclusion criteria for all participants were: (i) after two-dose vaccination (BBIBP-CorV or CoronaVac), (ii) age ≥ 18 years, key exclusion criteria were: (i) history of COVID-19 infection, (ii) autoimmune disease or use of immunosuppressants, and (iii) pregnancy. It is sure that all participants did not experience “white coat hypertension”.

All HTN patients were split into four groups for further analysis: secondary hypertension group (SH) (*n* = 16). Furthermore, essential hypertension will be classified into grades 1 to 3 depending on the grade in which patients received their hypertension diagnosis [essential grades 1 (EHPG1), 140 mmHg ≤ SBP < 159 mmHg or 90 mmHg ≤ DBP < 99 mmHg, n = 11, essential grades 2(EHPG2], 160 mmHg ≤ SBP < 179 mmHg or 100 mmHg ≤ DBP < 109 mmHg, *n* = 24, essential grades 3(EHPG3), SBP ≥ 180 mmHg or DBP ≥ 110 mmHg, *n* = 43).

All HTN patients were split into two subgroups according to with or without CCVD (CCVD: mainly include coronary atherosclerotic heart disease, hypertensive heart disease, cerebral infarction, and atherosclerosis): HTN with CCVD group (n=56) or without CCVD group (*n*=38).

To standardize the timing of vaccination (“1 month” was defined as 14–45 days, “2 months” as 46–75 days, “3 months” as 76–105 days, and over 3 months).

### Record of adverse events

A questionnaire was used to evaluate AEs 7 and 30 days after the 2nd dose of vaccine. The Chinese Medical and Drug Administration scale was then used to classify all AEs (2019 version).

### Antibody testing

S-RBD IgG and SARS-CoV-2 NAbs of blood samples were detected by MAGLUMI 2000 (Snibe, shenzhen, China). Based on the kit instructions, the threshold of anti-RBD IgG Abs which is >1.00 AU/ml is seropositive, and the threshold of CoV-2 NAbs that is >0.15 μg/ml is seropositive; on the contrary, less than or equal to the critical value is seronegative.

### Detection of SARS-CoV-2-specific B cells

The stained peripheral blood mononuclear cells were tested by Beckman flow cytometry (Beckman Coulter, Inc., California, USA). The specific steps were as follows: first, we mixed the biotinylated SARS-CoV-2 spike RBD protein (40592-V08H2-B, Sino biological, Beijing, China) with streptavidin-BV421 (405225, Biolegend, California, USA) in a 4:1 mole ratio and leave for 1 h to get the antigen probe; second, peripheral blood mononuclear cells were obtained from whole heparinized blood; the density gradient centrifugation was performed by Histopaque (10771, Sigma–Aldrich, St Louis, Missouri, USA) and then cleaned by cytometric staining buffer (FACS, 2%FBS) cells, besides we added antigen probe (1:33.3), fluorescence-coupled antibody [anti-human IgG Fc (410722, Biolegend), anti-human IgM (314524, Anti-human) CD3 (300430, Biolegend), anti-human CD19 (302212, Biolegend), anti-human CD21 (354918, Biolegend), and anti-human CD27 (356406, Biolegend)] into the cells and dyed at 4°C for 30 min under dark condition. After being re-suspended with FACS buffer, the samples were tested on the machine. The data were analyzed by Flow Jo software (V10.0.7).

### Statistical analysis

The median (range) test was used for ordinal variable analysis, and the chi-square test or Fisher's exact test was used for categorical variable analysis. Mann–Whitney U test and Kruskal–Wallis test were used for continuous variables. All results of multiple comparisons were corrected using Dunn's multiple comparisons test and Bonferroni. To examine the relationship between Abs and RBD-specific B cells, Spearman's rank correlation was used. The factors that significantly impacted Ab titers were identified using univariate and multivariate ordinal linear regression analyses. For statistical analysis, GraphPad Prism version 9.2.0 and SPSS 26 (IBM Corp., Armonk, NY, USA) were used. ^*^p < 0.05, ^**^p < 0.01, and ^***^p < 0.001.

## Results

### Participants' characteristics

The study cohort included 168 participants. As shown in Table 1, comparisons between the HTN patients and HCs found no significant differences in median age, percentage of men, mean body mass index, vaccine type, median number of days after 2nd vaccination, results of routine blood tests (white blood cells, hemoglobin, lymphocytes, and platelets), liver function markers (aspartate transaminase and alanine aminotransferase), and creatinine (Table 1).

**Table 1:** The general characteristics of HTN patients and HCs.

**Variables**	**HTN patients (*n* = 94)**	**HCs (*n* = 74)**	***p***
Age (years)	66 (19–89)	64 (30–87)	0.214
age ≥ 60years	67.0%(63/94)	60.8%(45/74)	0.404
Gender [male, *n* (%)]	51.1% (48/94)	6.8% (42/74)	0.463
Body mass index^#^ (Kg/m^2^)	24.2 (16.60–48.83)	23.7 (16.80–30.22)	0.641
Vaccine type(CoronaVac)	63.8%(60/94)	70.3%(52/74)	0.379
Days after 2^nd^ dose, median(range)	41 (16–168)	41.5 (18–142)	0.798
red blood cell^#^(10^∧12/*L*^)	4.40 (2.37–9.07)	4.54 (2.32–5.57)	0.381
white blood cell^#^(10^∧9/*L*^)	6.37 (2.95–19.08)	6.04 (3.11–11.07)	0.228
hemoglobin^#^(g/L)	136.5 (71–208)	38 (101–138)	0.492
lymphocyte^#^(10^∧9/*L*^)	1.60 (0.35–3.72)	1.70 (0.21–5.91)	0.269
platelet^#^(10^∧9/*L*^)	190 (58–1,182)	198 (101–420)	0.316
aspartate transaminase^#^ (IU/L)	20.0(3.0–82.0)	9.7(6.0–68.0)	0.787
alanine aminotransferase^#^(IU/L)	20.0(8.0–65.0)	21.0(10.0–48.0)	0.873
creatinine^#^ (μmol/L)	75.90 (36.3–1,458.4)	68.75 (1.80–735.6)	0.093

^#^Displayed as median (Range). For categorical variables, Fisher's exact test and the chi-square statistic test were utilized, while the Mann–Whitney U test was used for continuous variables.

### Vaccine safety

As shown in Table 2, after immunization, the incidence of negative outcomes after 7 days was comparable in HTN (8.5% vs. 9.5%, *p* = 0.830) compared with controls. The most frequent local adverse event was pain at the injection site, which occurred in 2.1% (2/94) of HTN patients and 4.1% (3/74) of HCs. The most frequent systemic adverse event in HTN patients was fever (2.1%), while the most frequent adverse event in healthy controls was weariness (4.1%). Self-reports of AEs of all participants indicated that they were all minor (grades 1 and 2). After extending the observation period to 30 days, only one additional occurrence of mild AEs—including one instance of pain at the injection site—was observed in HTN patients, and there were no new adverse events in the healthy controls.

**Table 2:** AEs of COVID-19 vaccination in participants.

**Variable**	**HTN patients**	**HCs**	***p***
Overall adverse events within 7 days	8(8.5%)	7(9.5%)	0.830
Overall adverse events within 30 days	9(9.6%)	7(9.5%)	0.980
**Local adverse events**			
Pain	2(2.1%)	3(4.1%)	0.785
Swelling	/	1(1.4%)	1.000
Itch	1(1.1%)	/	1.000
**Systemic adverse events**			
Fatigue	1(1.1%)	3(4.1%)	0.452
Drowsiness	1(1.1%)	2(2.7%)	0.832
dizziness	/	1(1.4%)	1.000
Fever	2(2.1%)	/	1.000
Cough	1(1.1%)	1(1.4%)	1.000
Gastro spasm	1(1.1%)	/	1.000
Decreased hemoglobin	/	/	1.000
Decreased platelet count	/	/	1.000
Decreased albumin	/	/	1.000
Elevated liver enzymes	/	/	1.000
Grade 3 and 4 adverse events	/	/	1.000

The data are expressed as n (%). Fisher's exact test and the chi-Square statistic test were used in comparison.

### Humoral immune response to inactivated SARS-CoV-2 vaccines in HTN

**Figure 1:**
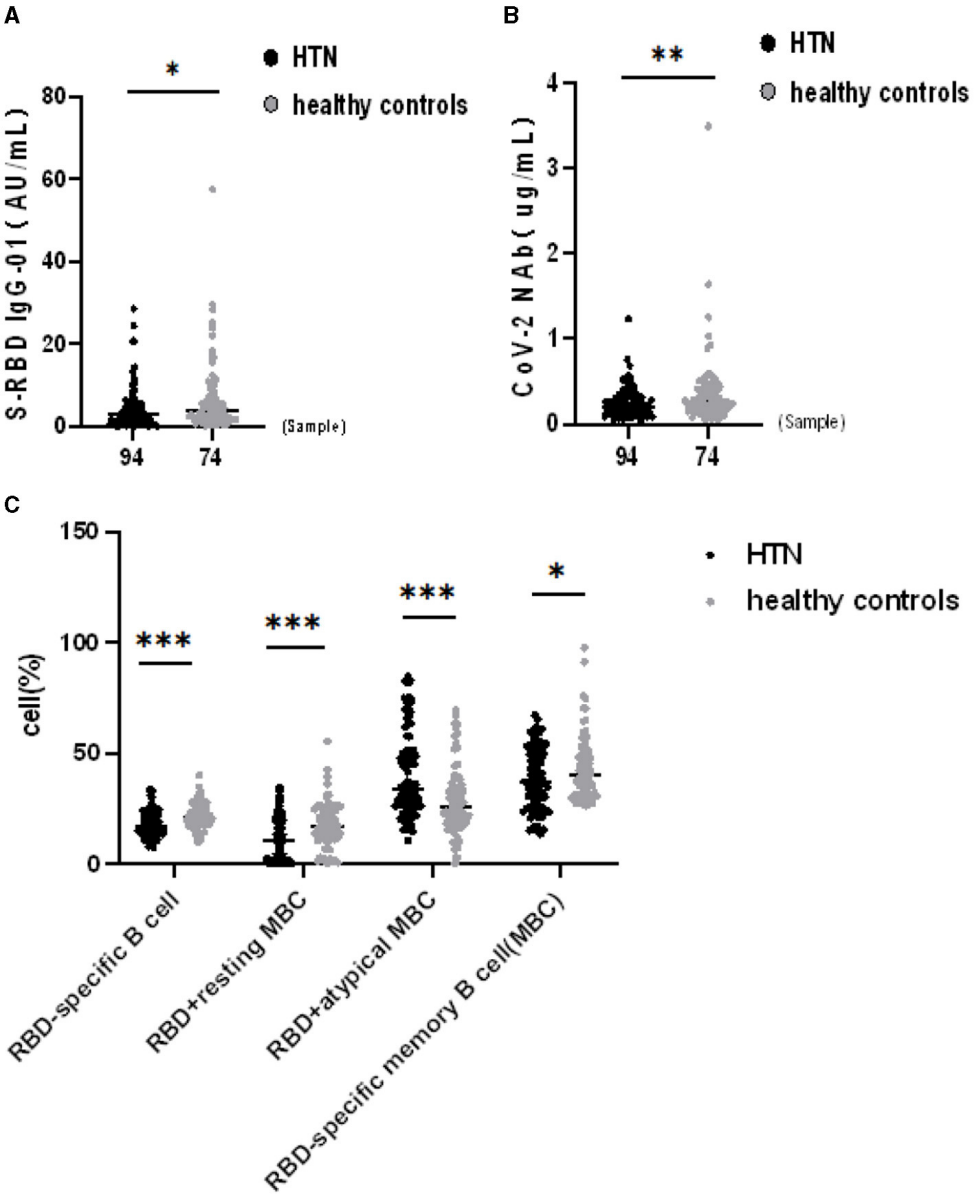
**(A–C)** Humoral immune responses to inactivated SARS-CoV-2 vaccines in HTN. *p < 0.05, **p < 0.01, ***p < 0.001.

The seropositivity rates of both Abs were lower in HTN patients than HCs in Table 3. The titers of both Abs in HTN patients were significantly lower than in HCs (median [IQR], 2.80 [0.86–5.46] vs. 3.73 [1.78–8.04], *p* = 0.020, 0.21 [0.12–0.34] vs. 0.26 [0.18–0.44], *p* = 0.009, respectively) (Figures 1A,B). HTN patients were lower the frequencies of RBD-specific B cells, RBD^+^ resting MBCs, and RBD-specific MBCs compared with HCs, but higher frequencies of RBD^+^ atypical MBCs compared with HCs(median [IQR], 17.05 [13.58–22.20] vs. 21.05 [17.60–24.53], *p* = 0.001, 10.20 [1.52–19.63] vs. 17.10 [11.88–24.28], *p* = 0.001, and 37.10 [25.35–51.20] vs. 40.25 [31.70–50.05], *p* = 0.048, 33.70 [25.95–50.43] vs. 25.65 [18.48–35.93], *p* = 0.001, respectively) (Figure 1C).

**Table 3:** The seropositivity rates of both Abs in HTN and HCs.

**Seropositivity rates(%)**	**HTN patients (94)**	**HCs (74)**	***P***
S-RBD IgG	72.34% (68/94)	91.89% (68/74)	0.001
CoV-2 NAb	63.83% (60/94)	82.43% (61/74)	0.008

RBD, receptor binding domain, the chi-square statistic test was used in comparison.

### Humoral immune response to inactivated SARS-CoV-2 vaccines in HTN subgroups

Following that, we discovered that the seropositivity rates of both Abs were similar in SH, EHPG1, 2, and 3 (Table 4).

**Table 4:** The seropositivity rates of both Abs in HTN subgroups.

**Seropositivity rates (%)**	**SH (16)**	**EHPG1 (11)**	**EHPG2 (24)**	**EHPG3 (43)**	***P***
S-RBD IgG	62.5% (10/16)	81.8% (9/11)	58.3% (14/24)	79.1% (34/43)	0.218
CoV-2 NAb	62.5% (10/16)	81.8% (9/11)	50% (12/24)	67.4% (29/43)	0.256

RBD, receptor binding domain, the chi-square, Fisher's exact test, and Dunn's multiple comparison tests were used for comparisons.

**Figure 2:**
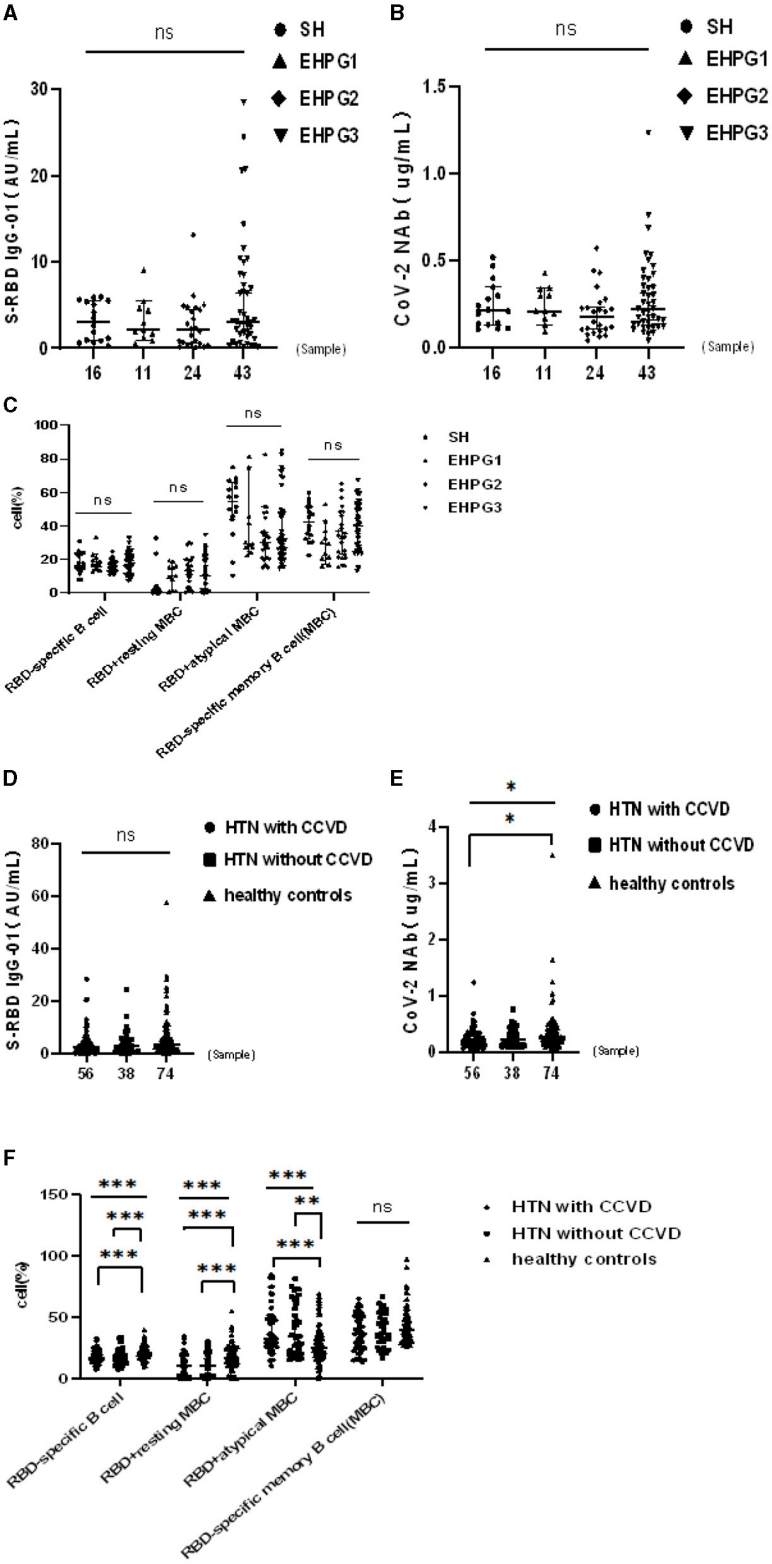
**(A–F)** Humoral immune responses to inactivated SARS-CoV-2 vaccines in HTN subgroups. *p < 0.05, **p < 0.01, ***p < 0.001.

In a subsequent research, the seropositivity rates of anti-RBD IgG and CoV-2 NAbs in SH, EHPG1, 2, and 3 showed no difference after analysis (68.8% vs. 81.8% vs. 58.3% vs. 79.1%, *p* = 0.280 and 62.5% vs. 81.8% vs. 50.0% vs. 67.4%, *p* = 0.274, respectively). The titers of anti-RBD IgG and CoV-2 NAbs were similar to SH, EHPG1, 2, and 3 (3.06 [0.88–5.48] vs. 2.17 [1.21–4.63] vs. 2.12 [0.56–4.61] vs. 3.07 [1.40–8.43], *p* = 0.284 and 0.21 [0.13–0.34] vs. 0.21 [0.17–0.34] vs. 0.18 [0.10–0.25] vs. 0.22 [0.14–0.39], *p* = 0.258, respectively) (Figures 2A,B).

However, there was no difference in the frequencies of RBD-specific B cells, RBD^+^ resting MBCs, RBD^+^ atypical MBCs, and RBD-specific memory B cells (MBCs) of SH or EHPG 1, 2, or 3 (Figure 2C).

Following that, we discovered that the seropositivity rates of both Abs were lower in HTN patients with or without CCVD than HCs in Table 5. Moreover, the titers of CoV-2 NAb were significantly lower in HTN patients with CCVD than HCs (median [IQR], 0.20 [0.19–0.30] vs. 0.26 [0.28–0.49], *p* = 0.019) (Figures 2D,E). The frequencies of RBD-specific MBCs were somewhat lower in HTN patients with or without CCVD than HCs, even if there was no statistical difference (Figure 2F).

**Table 5:** The seropositivity rates of both Abs in HTN subgroups and HCs.

**Seropositivity rates (%)**	**HTN with CCVD (56)**	**HTN without CCVD (38)**	**HCs (74)**	***P***
S-RBD IgG	^**^71.43% (40/56)	^##^73.68% (28/38)	91.89% (68/94)	0.006
CoV-2 NAb	^*^64.29% (36/56)	^#^63.16% (24/38)	82.43% (61/74)	0.028

RBD, receptor binding domain, the chi-square, Fisher's exact, and Dunn's multiple comparisons tests were used for comparisons (^*^p < 0.05, ^**^p < 0.01 vs. HCs, ^#^p < 0.05, ^##^p < 0.01 vs. HCs).

### Humoral immune to inactivated SARS-CoV-2 vaccines in HTN by age

**Figure 3:**
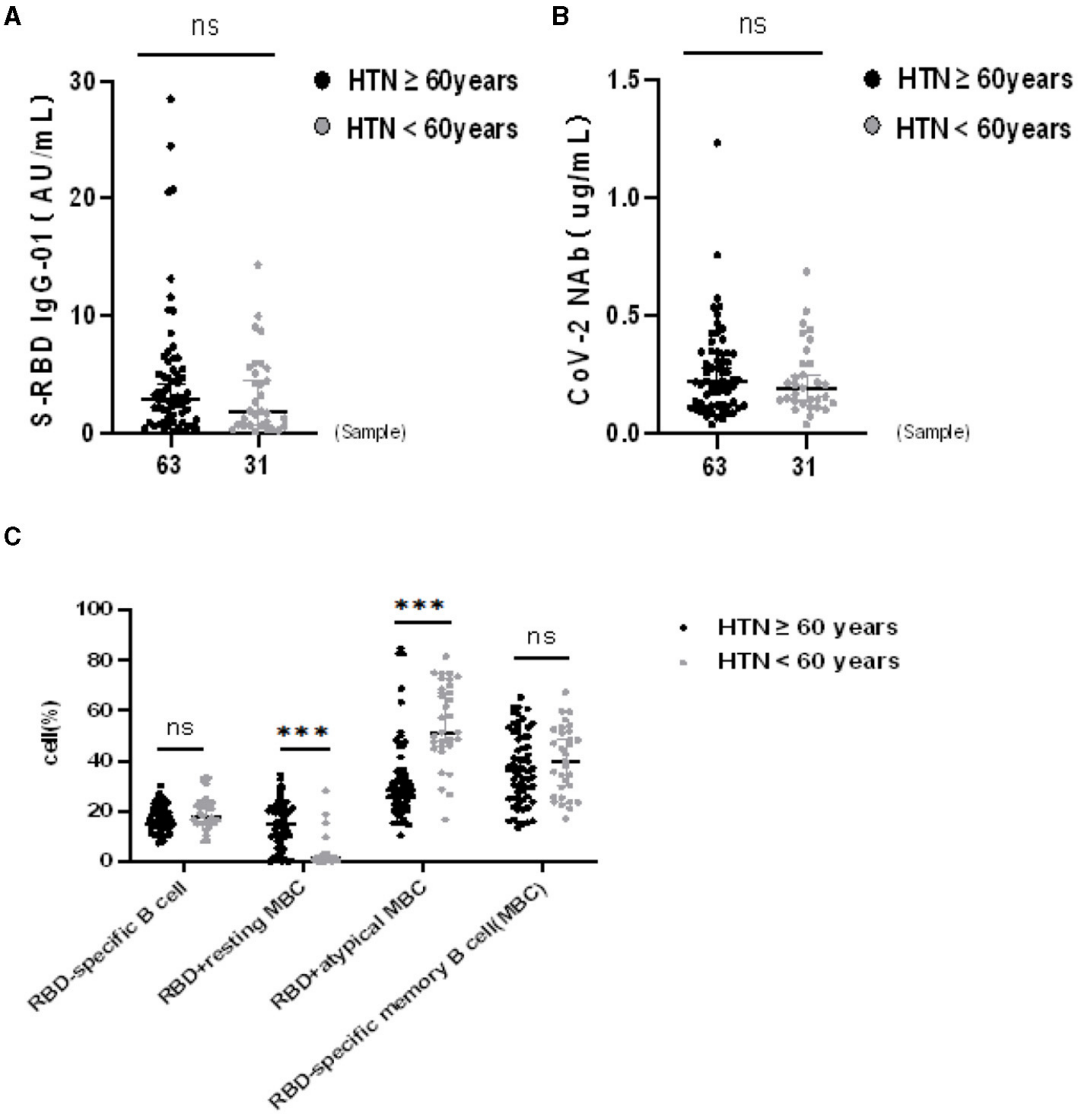
**(A–C)** Humoral immune responses to inactivated SARS-CoV-2 vaccines in HTN by age. ***p < 0.001.

The seropositivity rates of both Abs were similar in HTN patients aged ≥ 60 and < 60 years, as shown in Table 6. Moreover the titers of both Abs were similar in HTN patients aged ≥ 60 and < 60 years (median[IQR], 2.90 [1.09–5.44] vs. 1.84 [0.60–5.52], *p* = 0.216 and 0.22 [0.12–0.34] vs. 0.19 [0.13–0.30], *p* = 0.628, respectively) (Figures 3A,B). The frequencies of RBD^+^ resting MBCs were higher in HTN patients aged ≥60 and < 60 years, and the frequencies of RBD^+^ atypical MBCs were lower in HTN patients aged ≥60 and < 60 years (median [IQR], 15.10 [8.38–21.10] vs. 1.39 [0.69–2.46], *p* = 0.000) and 28.70 [21.70–36.00] vs. 51.40 [45.90–69.90], *p* = 0.000, respectively) (Figure 3C).

**Table 6:** The seropositivity rates of both Abs in HTN by age.

**Seropositivity rates(%)**	**HTN ≥60 years (63)**	**HTN < 60 years (31)**	***P***
S-RBD IgG	76.19% (48/63)	67.74% (21/31)	0.383
CoV-2 NAb	66.67% (42/63)	58.06% (18/31)	0.414

RBD, receptor binding domain, the chi-square statistic test was used for comparison.

### Humoral immune responses to inactivated SARS-CoV-2 vaccines in HTN over time

**Figure 4:**
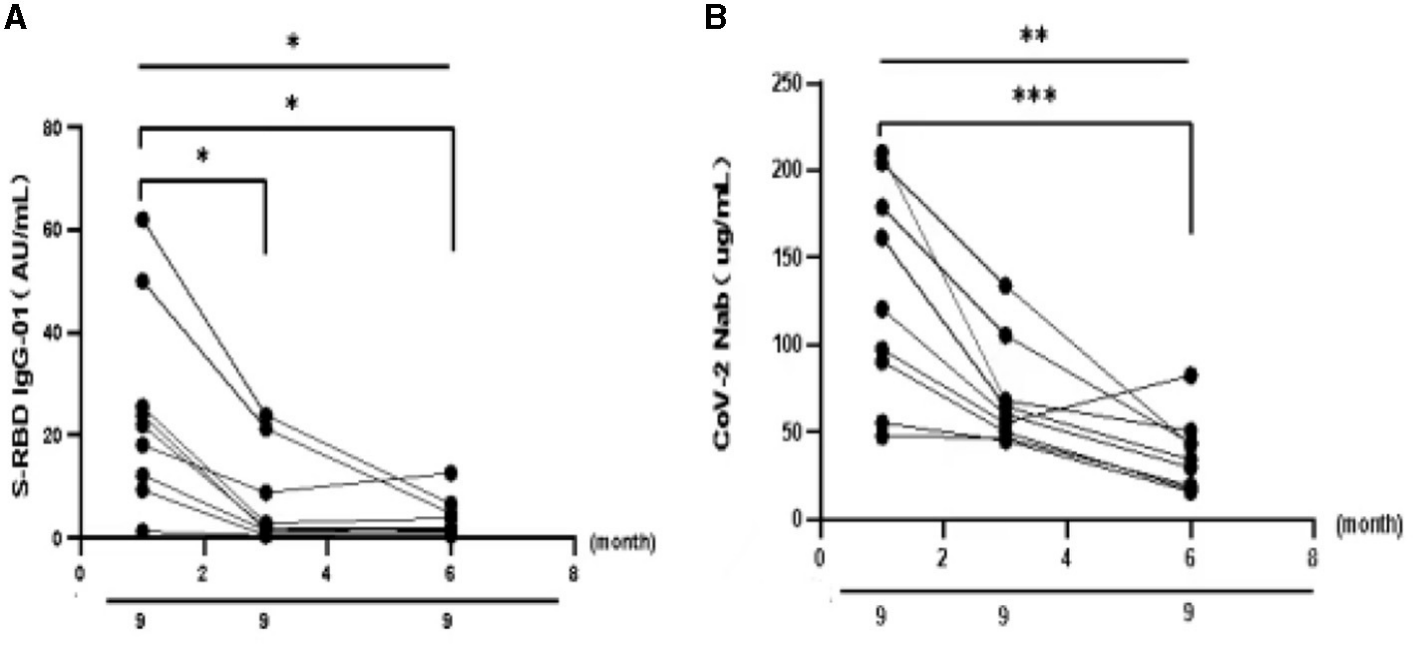
**(A, B)** The titers of both Abs in HTN over time. *p < 0.05, **p < 0.01, ***p < 0.001.

**Figure 5:**
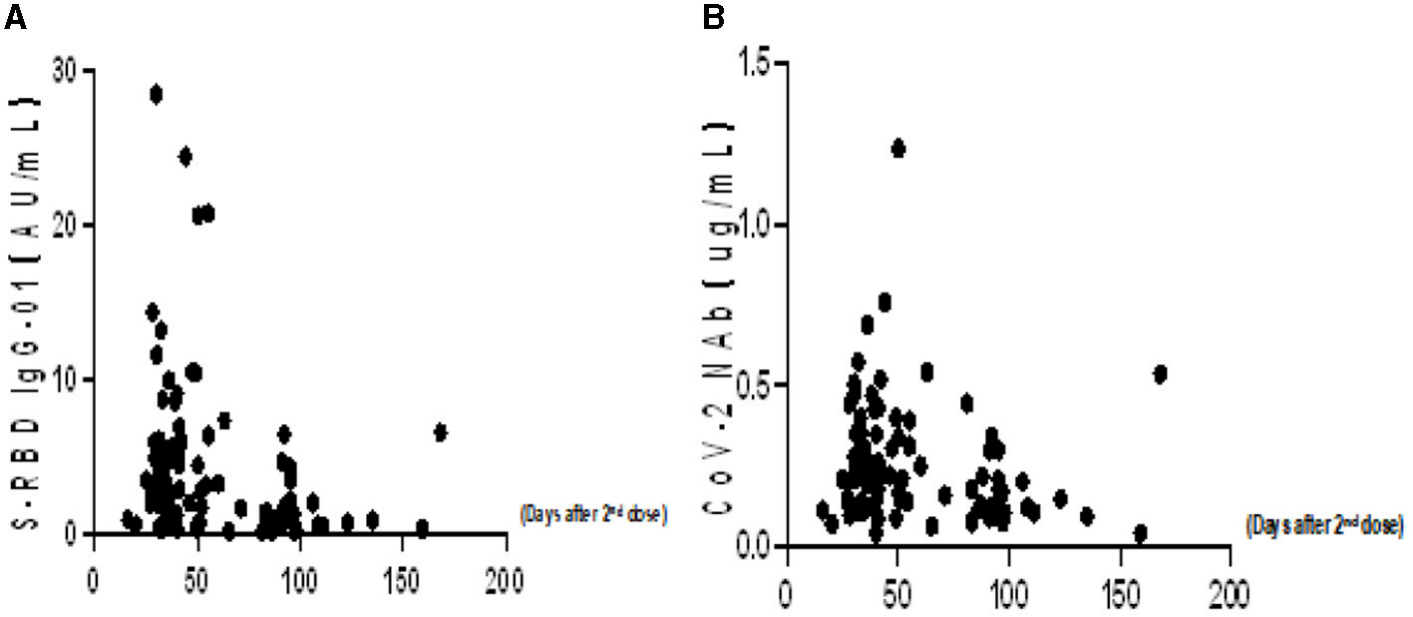
**(A, B)** The titers of both Abs in HTN over time (days).

To better understand the variation of humoral immune responses with passing time, we followed up some HTN patients. As expected, the seropositivity rates and titers of both Abs gradually decreased over time in HTN patients, especially from 1 month to 3 months (Table 7, Figures 4A,B). The titers of both Abs were declined with the days after the second dose in HTN patients (Figures 5A,B).

**Table 7:** The seropositivity rates of both Abs in HTN over time.

**Seropositivity rates(%)**	**1 month**	**3 month**	**6 month**
S-RBD IgG	^*^88.89% (8/9)	33.33% (3/9)	33.33%(3/9)
CoV-2 NAb	^**^77.78% (7/9)	11.11% (1/9)	/(0/9)

RBD, receptor binding domain, the chi-square, Fisher's exact, and Dunn's multiple comparisons tests were used for comparisons (^*^p < 0.05, ^**^p < 0.01 vs. 3 month).

Finally, we are interested in learning more about the variables that affect anti-RBD IgG Abs and CoV-2 NAbs response in HTN patients. The time period following a two-dose vaccination was the key element in Tables 8, 9 that was connected to a poor Ab response.

**Table 8:** Univariate and multivariate analyses for anti-RBD IgG Abs in HTN.

	**Univariate OR (95%CI)**	***p***	**Multivariate OR (95%CI)**	***p***
Gender (female)	0.733 (0.295, 1.382)	0.212	0.808 (0.236, 2.764)	0.734
Age (years)	1.000 (0.966, 1.035)	0.960	0.958 (0.903, 1.016)	0.149
Body mass index (Kg/m∧2)	1.014 (0.914, 1.141)	0.804	1.043 (0.919, 1.183)	0.512
Days after 2nd dose	0.976 (0.961, 0.991)	0.002	0.959 (0.938, 0.979)	< 0.001
Vaccine tape (Corona Vac)	1.792 (0.707, 4.537)	0.216	0.992 (0.302, 3.260)	0.990
Red blood cell (10^∧12/*L*^)	1.300 (0.732, 2.502)	0.402		
Hemoglobin (g/L)	1.015 (0.993, 1.039)	0.189		
white blood cell (10^∧9/*L*^)	1.088 (0.867, 1.433)	0.507		
Lymphocyte (10^∧9/*L*^)	1.151 (0.546, 2.551)	0.717		
Platelet (10^∧9/*L*^)	1.001 (0.997, 1.007)	0.676		
Aspartate transaminase (IU/L)	1.022 (0.987, 1.066)	0.254		
Alanine aminotransferase (IU/L)	1.007 (0.963, 1.060)	0.254		
Creatinine (μmol/L)	1.000 (0.998, 1.001)	0.480		
EHPG1	0.687 (0.192, 2.457)	0.564	0.191 (0.026, 1.405)	0.104
EHPG2	3.437 (0.394, 30.009)	0.264	3.253 (0.168, 63.084)	0.435
EHPG3	0.437 (0.149, 1.286)	0.133	0.111 (0.021, 0.589)	0.010
HTN without CCVD	0.712 (0.278, 1.823)	0.479	0.980 (0.282, 3.407)	0.975
RBD-specific B cells (*n* %)	1.034 (0.956, 1.125)	0.413	1.041 (0.931, 1.165)	0.478
RBD^+^ resting MBCs (*n* %)	1.051 (1.000, 1.111)	0.061	1.049 (0.944, 1.166)	0.374
RBD^+^ atypical MBCs (*n* %)	0.985 (0.962, 1.008)	0.197	0.968 (0.909, 1.031)	0.313
RBD-specific MBCs (*n* %)	0.995 (0.963, 1.028)	0.762	0.957 (0.904, 1.014)	0.137

MBC, memory B cell; CI, confidential interval; OR, odds ratio; RBD, receptor binding domain.

**Table 9:** Univariate and multivariate analyses for CoV-2 NAbs in HTN.

	**Univariate OR (95%CI)**	***p***	**Multivariate OR (95%CI)**	***p***
Gender (female)	0.739 (0.314, 1.716)	0.482	0.966 (0.349, 2.678)	0.948
Age (years)	1.004 (0.972, 1.035)	0.828	0.979 (0.934, 1.027)	0.394
body mass index (Kg/m^2^)	1.062 (0.958, 1.197)	0.289	1.070 (0.952, 1.203)	0.256
Days after 2nd dose	0.986 (0.972, 0.999)	0.049	0.976 (0.959, 0.994)	0.008
Vaccine tape (CoronaVac)	1.704 (0.713, 4.084)	0.229	1.173 (0.421, 3.270)	0.760
red blood cell (10^∧12/*L*^)	1.067 (0.632, 1.875)	0.809		
Hemoglobin (g/L)	1.015 (0.994, 1.037)	0.184		
white blood cell (10^∧9/*L*^)	1.014 (0.824, 1.274)	0.896		
Lymphocyte (10^∧9/*L*^)	1.239 (0.615, 2.596)	0.556		
Platelet (10^∧9/*L*^)	0.997 (0.991, 1.001)	0.240		
aspartate transaminase (IU/L)	1.021 (0.989, 1.060)	0.226		
alanine aminotransferase (IU/L)	1.030 (0.985, 1.086)	0.226		
Creatinine (μmol/L)	1.000 (0.998, 1.000)	0.267		
EHPG1	0.926 (0.281, 3.052)	0.899	0.744 (0.157, 3.524)	0.709
EHPG2	6.111 (0.717, 52.056)	0.098	8.799 (0.718, 107.885)	0.089
EHPG3	0.867 (0.201, 1.539)	0.258	0.300 (0.079, 1.143)	0.078
HTN without CCVD	0.867 (0.366, 2.051)	0.479	1.374 (0.475, 3.976)	0.557
RBD-specific B cells (*n* %)	1.020 (0.949, 1.100)	0.594	1.018 (0.931, 1.112)	0.699
RBD^+^ resting MBCs (*n* %)	1.053 (1.005, 1.107)	0.034	1.076 (0.981, 1.180)	0.120
RBD^+^ atypical MBCs (*n* %)	0.985 (0.963, 1.007)	0.174	0.994 (0.9441, 1.052)	0.802
RBD-specific MBCs (*n* %)	1.004 (0.974, 1.035)	0.801	0.992 (0.954, 1.031)	0.689

MBC, memory B cell; CI, confidential interval; OR, odds ratio; RBD, receptor binding domain.

Analysis revealed a negative connection between the titers of anti-RBD IgG Abs and RBD^+^ atypical MBCs in all participants (R = −0.168, *p* = 0.030) (Table 10).

**Table 10:** The correlation between Ab titers and RBD-specific MBCs in participants.

	**S-RBD IgG (AU/ml)**		**CoV-2 NAb (ug/ml)**	
	**R**	***p***	**R**	***p***
RBD-specific B cells (*n* %)	0.035	0.655	0.109	0.161
RBD^+^ resting MBCs (*n* %)	0.152	0.050	0.137	0.077
RBD^+^ atypical MBCs (*n* %)	−0.168	0.030	−0.128	0.098
RBD-specific MBCs (*n* %)	−0.024	0.757	−0.014	0.854

R, relative; MBC, memory B cell; RBD, receptor binding domain.

## Discussion

In this prospective investigation, the safety, Abs response, RBD-specific B cells, and MBCs were evaluated between HTN patients and healthy controls for inactivated SARS-CoV-2 vaccinations. Inactivated vaccinations were well tolerated in HTN, according to our findings. In HTN, both Abs and RBD-specific MBC responses were limited.

According to the most recent data from the Chinese Hypertension Survey, the prevalence of hypertension in people aged 18 years and older in China was 27.9% between 2012 and 2015 (standardized rate: 23.2%) (16) and showed a tendency of rising annually.

In HTN with infected COVID-19, the mortality rate rose (4, 17–19). However, there are just a few safety and immunogenicity trials on inactivated SARS-CoV-2 vaccinations in populations with hypertension. As a result, we initially evaluated the safety of inactivated vaccinations in HTN. We discovered that the overall incidence of adverse events within 7 days and 30 days of vaccination was similar in HTN patients and HCs, respectively, (8.5% vs. 9.5%) and (9.6% vs. 9.5%). However, this finding is significantly lower than that of previous studies, such as phase 1/2 trials of BBIBP-CorV in China (23–29%) (20) and phase 3 trials of Corona Vac in Turkey (18.9%) (21).

Recent data suggest that immune system malfunction may be the cause of hypertension and a subpar immunization response (22). After receiving the SARS-CoV-2 vaccine, the production and maintenance of antigen-specific isotype-switched memory B cells and high-affinity neutralizing antibodies are crucial for the preservation of protective humoral immunity (23). Our findings showed that following immunization, the titers of anti-RBD IgG Abs and CoV-2 NAbs in HTN patients were lower than HCs, which is consistent with earlier research, revealing that these patients had less favorable immune responses to the vaccine (3, 9, 24). Although the titers of anti-RBD IgG Abs were lower in HTN than HCs, the frequencies of RBD^+^ atypical MBCs were higher in HTN. We found that the titers of anti-RBD IgG Abs were adversely linked with the frequencies of RBD^+^ atypical MBCs by correlation analysis. Atypical memory B cells are described as fatigued B cells that are inhibiting the production of Abs; however, they typically occur in great numbers in chronic diseases (25–27). Therefore, it is suggested that a possible correlation between an increase in the frequencies of RBD^+^ and atypical MBC frequencies reduced the titers of anti-RBD IgG Abs.

MBCs are necessary for sustaining long-lasting humoral immunity to pathogenic agents (28). Previous research has demonstrated that RBD-specific MBCs can survive in a vaccinated person for an extended period of time, up to 1 year (29, 30). Another investigation verified that the MBC repertoire produced by mRNA vaccines still gives some protection against the Omicron variant in people who have received vaccination, despite the Omicron variant has a considerable immune escape potential (31). We therefore focus on how MBCs react to inactivated vaccination. In this investigation, RBD-specific MBCs can still be evaluated in HTN patients and HCs after 6 months of receiving the whole course of immunization. Additionally, we discovered that HTN had lower frequencies of RBD-specific MBCs than HCs, which is consistent with earlier research (21) and showed that the diminished humoral immunity in HTN patients may be brought on by an immune system that is not functioning properly. In this investigation, through subgroup analysis, we discovered that the titers of CoV-2 NAbs were lower in HTN patients with CCVD than in healthy controls; in contrast, this result was not found in HTN patients without CCVD. Analysis of the reason may be that the humoral immunity level of these people is low or our sample size was small. We found that the titers of both Abs were similar among SH, EHPG1, EHPG2, and EHPG3; therefore, we hypothesized that patients with different grades of hypertension may experience comparable immunogenicity.

Age is a significant risk factor for hypertension as we are all aware. Previous research has demonstrated that the immunogenicity of the COVID-19 BNT162b2 vaccine results in a weaker antibody response in elderly than in young people (32), and that for people ages > 55 years, the total immune response is low, following a two-dose CoronaVac vaccination (33). However, in clinical trials, the Ab responses of elderly to the mRNA-1273 COVID-19 vaccination and the ChAdOx1nCoV-19 vaccine were comparable with that of young people (34, 35). When the Ab responses in HTN patients ages > 60 were evaluated, it was discovered that these patients were similar levels of Ab responses following two doses of the CoronaVac vaccine, and the audience and the type of vaccine may be to blame for this variation. Therefore, additional real-world data will be required in the future to confirm this. Through univariate and multivariate analyses, we found that the time period, following full-course vaccination, was a factor strongly connected to the levels of both Abs, which is similar to many other studies (36, 37). This implies that Ab titers in inactivated vaccines decreased with time; hence, we might need to deliver a booster dose to maintain Ab levels.

There were some restrictions in the current investigation. First of all, this was a single-center observation research in southwest China with a somewhat modest sample size. Second, T cells of the participants were not detected in this study due to experimental flaws. Third, only a few participants in this study were followed longitudinally. Fourth, antihypertensive drug regimens were not collected for hypertensive patients. Fifth, it is essential to acknowledge that individuals with hypertension may have other conditions, such as diabetes, dyslipidaemia, cardiac, and kidney problems, which could influence the immune response. However, this study also has some benefits: first, HTN patients in China were used to study the immunogenicity of SARS-CoV-2 vaccination. Second, this study thoroughly assessed humoral immune response to SARS-CoV-2. Third, it was once more established that subpar anti-RBD IgG Ab responses were related to the period of time, following the complete vaccination.

In conclusion, the inactivated SARS-CoV-2 vaccine was well tolerated among individuals with hypertension; however, humoral immune response was limited, especially merged CCVD, and declined gradually over time.

## Data availability statement

The raw data supporting the conclusions of this article will be made available by the authors, without undue reservation.

## Ethics statement

The studies involving humans were approved by the Second Affiliated Hospital, Chongqing Medical University. The studies were conducted in accordance with the local legislation and institutional requirements. The participants provided their written informed consent to participate in this study.

## Author contributions

LY: Writing – original draft. DJ: Writing – review & editing. TZ: Data collection, Writing – review & editing. YL: Data collection, Writing – review & editing. QG: Data collection, Writing – review & editing.
